# Supplementary material for: Components of a Neanderthal gut microbiome recovered from fecal sediments from El Salt
Source: Commun Biol. 2021 Feb 5;4:169. doi: 10.1038/s42003-021-01689-y (PMC7864912; doi:10.1038/s42003-021-01689-y)
Supplement: Supplementary file 2 — Supplementary Information [file 42003_2021_1689_MOESM2_ESM.pdf]

Supplementary Information for:
Components of a Neanderthal gut microbiome recovered from fecal sediments from El Salt

Simone Rampelli[†], Silvia Turroni[†], Carolina Mallol, Cristo Hernandez, Bertila Galván, Ainara Sistiaga, Elena Biagi, Annalisa Astolfi, Patrizia Brigidi, Stefano Benazzi, Cecil M. Lewis Jr., Christina Warinner, Courtney A. Hofman, Stephanie L. Schnorr[§], Marco Candela[§]

[†]These authors contributed equally.
[§]These authors jointly supervised this work.

This file includes:

- Supplementary Figure 1 to 9 p. 2 – 14
- Supplementary Table 1 to 3 p. 15 – 17
- Supplementary References p. 18

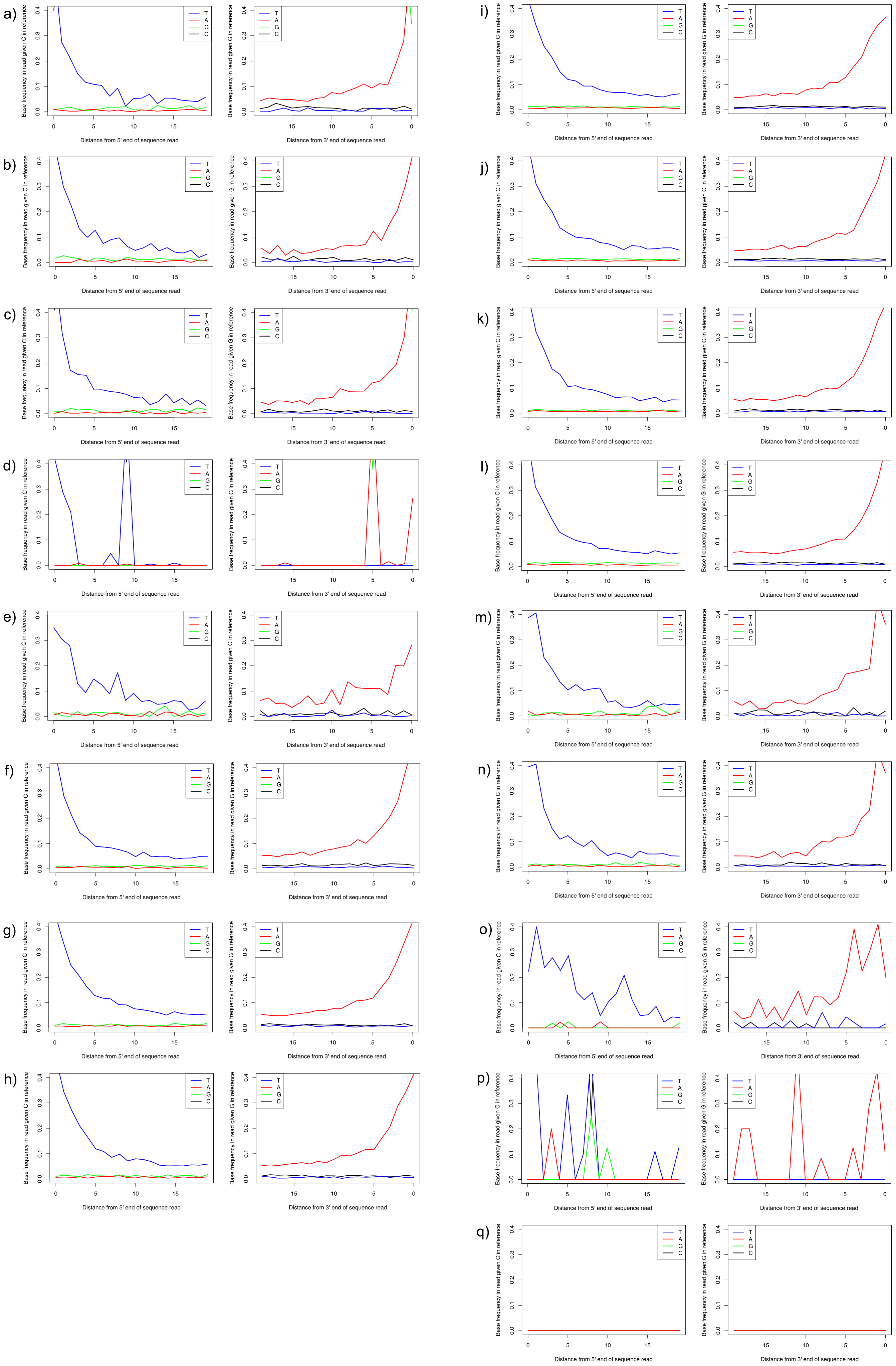
a)
b)
c)
d)
e)
f)
g)
h)
i)
j)
k)
l)
m)
n)
o)
p)
q)
Base frequency in read given C in reference
Base frequency in read given G in reference
Distance from 5' end of sequence read
Distance from 3' end of sequence read
T
A
G
C

Supplementary Figure 1.
Nucleotide mis-incorporation patterns arising from cytosine deamination in ancient bacterial DNA from El Salt sediment samples. Patterns are shown for all samples (**a** VI; **b** V2; **c** V3; **d** IX; **e** Xa; **f-l** ES1 to ES7; **m** Xb; **n** XI) as well as for negative controls (**o** DNA extraction; **p** library preparation; **q** PCR). Please refer to Supplementary Table 1 for the number of ancient bacterial reads of each sample.

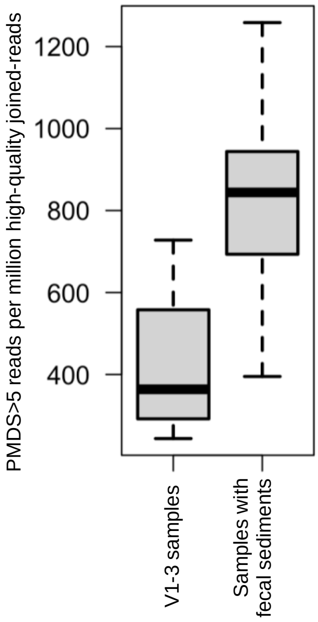

Supplementary Figure 2.
The proportion of PMDS>5 reads discriminates samples containing fecal sediment from those with no or very few archeological remains. The samples IX, Xa, ES1-7, Xb and XI (i.e., those positive for the presence of fecal biomarkers and/or associated with rich archaeological assemblages) were compared with samples V1-3 from SU V (i.e., negative samples, with no or very few archeological remains). P-value=0.01, Wilcoxon test.

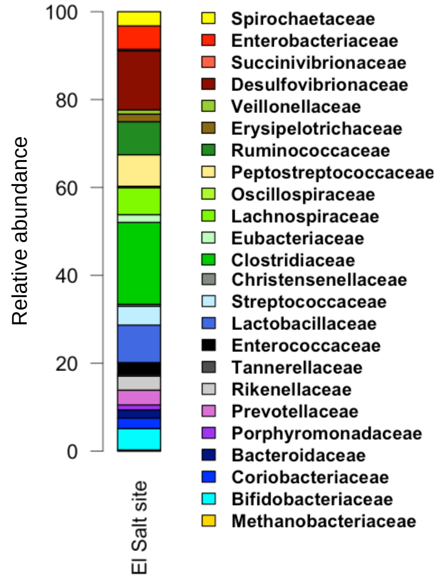

Supplementary Figure 3.
Bar plot of the relative abundance of the 24 families common to the gut microbiome of hominids in the El Salt samples. Comprehensive family-level relative abundance profile from all sedimentary samples positive for the presence of fecal biomarkers and/or associated with rich archaeological assemblages, i.e., samples IX, Xa, ES1-7, Xb and XI.

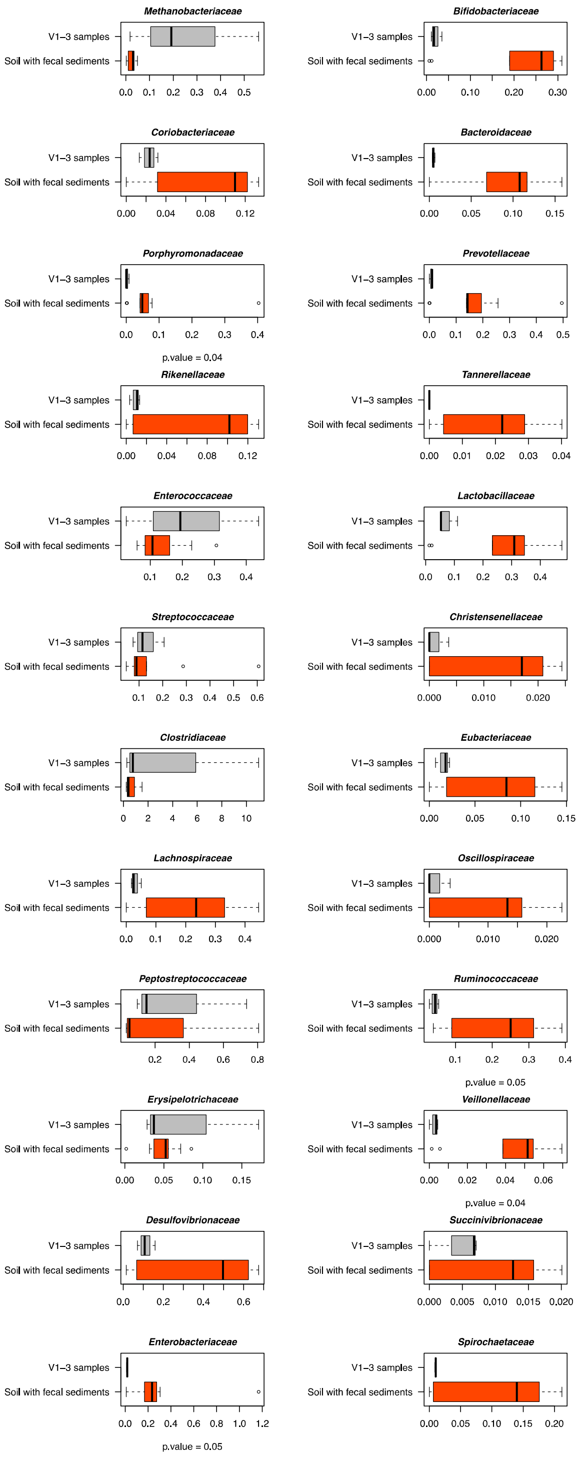

Supplementary Figure 4.

Box plots of the relative abundance of the 24 families common to the gut microbiome of hominids in the El Salt samples. Samples positive for the presence of fecal biomarkers and/or associated with rich archaeological assemblages (samples IX, Xa, ES1-7, Xb and XI) showed a higher relative contribution of PMDS>5 reads for most of the 24 families common to the gut microbiome of hominids compared to samples with no or very few archeological remains (samples from SU V). Relative abundances were calculated as a percentage of PMDS>5 reads for a specific family out of the total number of PMDS>5 reads in the sample. P-values ≤0.05 are reported (Wilcoxon test).

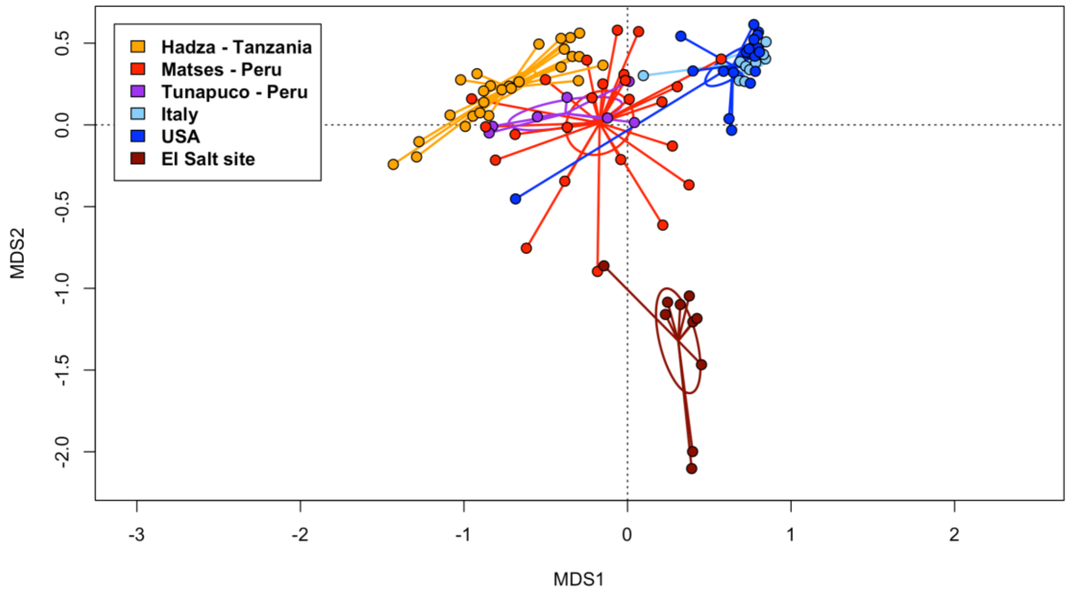

Supplementary Figure 5.

Principal Coordinates Analysis based on Bray-Curtis distances between the family-level relative abundance profiles of El Salt samples and the gut microbiota of human populations adhering to different subsistence strategies. The 24 families common to the gut microbiome of hominids were considered. For the El Salt site, samples IX, Xa, ES1-7, Xb and XI were included, i.e., those positive for the presence of fecal biomarkers and/or associated with rich archaeological assemblages. Publicly available data from the following human populations were included: urban Italians and Hadza hunter-gatherers from Tanzania (Rampelli *et al.*, 2015), urban US residents, Matses hunter-gatherers and Tunapuco rural agriculturalists from Peru (Obregon-Tito *et al.*, 2015). PCo1 and PCo2 account for 27% and 20% of the total variance, respectively. A significant separation between groups was found (p-value=0.0001, permutation test with pseudo-F ratio).

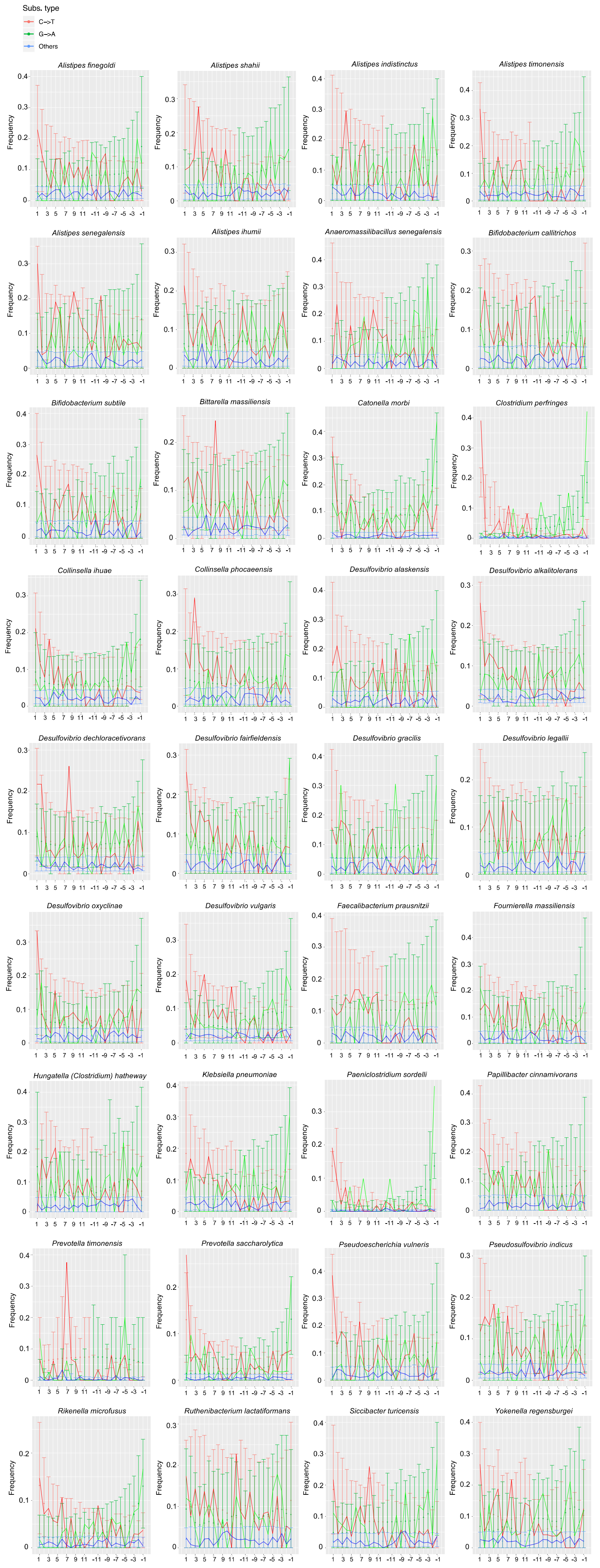
Subs. type
C-->T
G-->A
Others
Frequency
Alistipes finegoldi
Alistipes shahii
Alistipes indistinctus
Alistipes timonensis
Alistipes senegalensis
Alistipes ihumii
Anaeromassilibacillus senegalensis
Bifidobacterium callitrichos
Bifidobacterium subtile
Bittarella massiliensis
Catonella morbi
Clostridium perfringes
Collinsella ihuae
Collinsella phocaeensis
Desulfovibrio alaskensis
Desulfovibrio alkalitolerans
Desulfovibrio dechloracetivorans
Desulfovibrio fairfieldensis
Desulfovibrio gracilis
Desulfovibrio legallii
Desulfovibrio oxyclinae
Desulfovibrio vulgaris
Faecalibacterium prausnitzii
Fournierella massiliensis
Hungatella (Clostridium) hatheway
Klebsiella pneumoniae
Paeniclostridium sordelli
Papillibacter cinnamivorans
Prevotella timonensis
Prevotella saccharolytica
Pseudoescherichia vulneris
Pseudosulfovibrio indicus
Rikenella microfusus
Ruthenibacterium lactatiformans
Siccibacter turicensis
Yokenella regensburgei

Supplementary Figure 6.
MapDamage plots for bacterial taxa with ≥200 assigned reads and >50 reads with PMDS >1 recovered from the El Salt sediments.

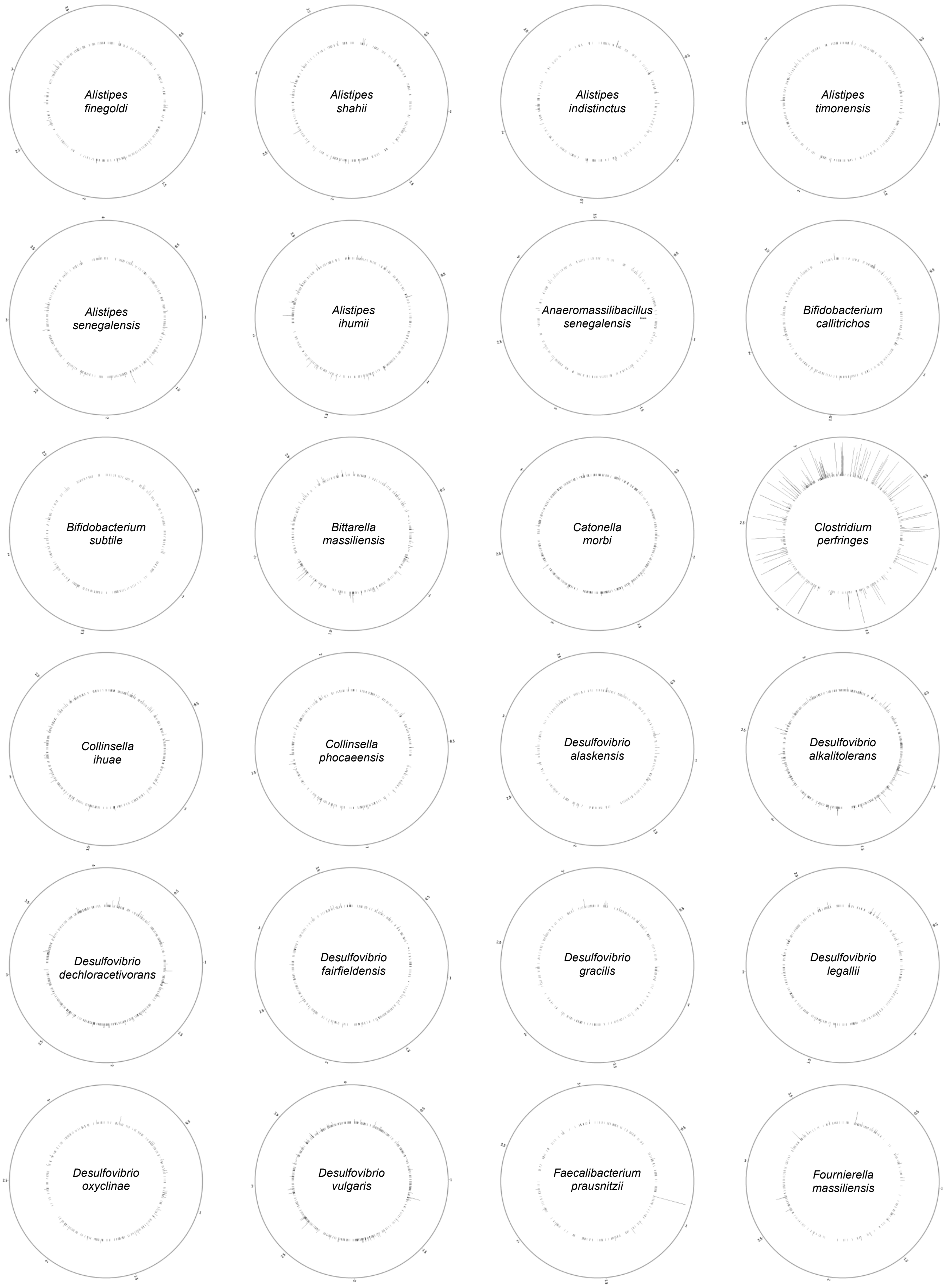
Alistipes finegoldi
Alistipes shahii
Alistipes indistinctus
Alistipes timonensis
Alistipes senegalensis
Alistipes ihumii
Anaeromassilibacillus senegalensis
Bifidobacterium callitrichos
Bifidobacterium subtile
Bittarella massiliensis
Catonella morbi
Clostridium perfringes
Collinsella ihuae
Collinsella phocaeensis
Desulfovibrio alaskensis
Desulfovibrio alkalitolerans
Desulfovibrio dechloracetivorans
Desulfovibrio fairfieldensis
Desulfovibrio gracilis
Desulfovibrio legallii
Desulfovibrio oxyclinae
Desulfovibrio vulgaris
Faecalibacterium prausnitzii
Fournierella massiliensis

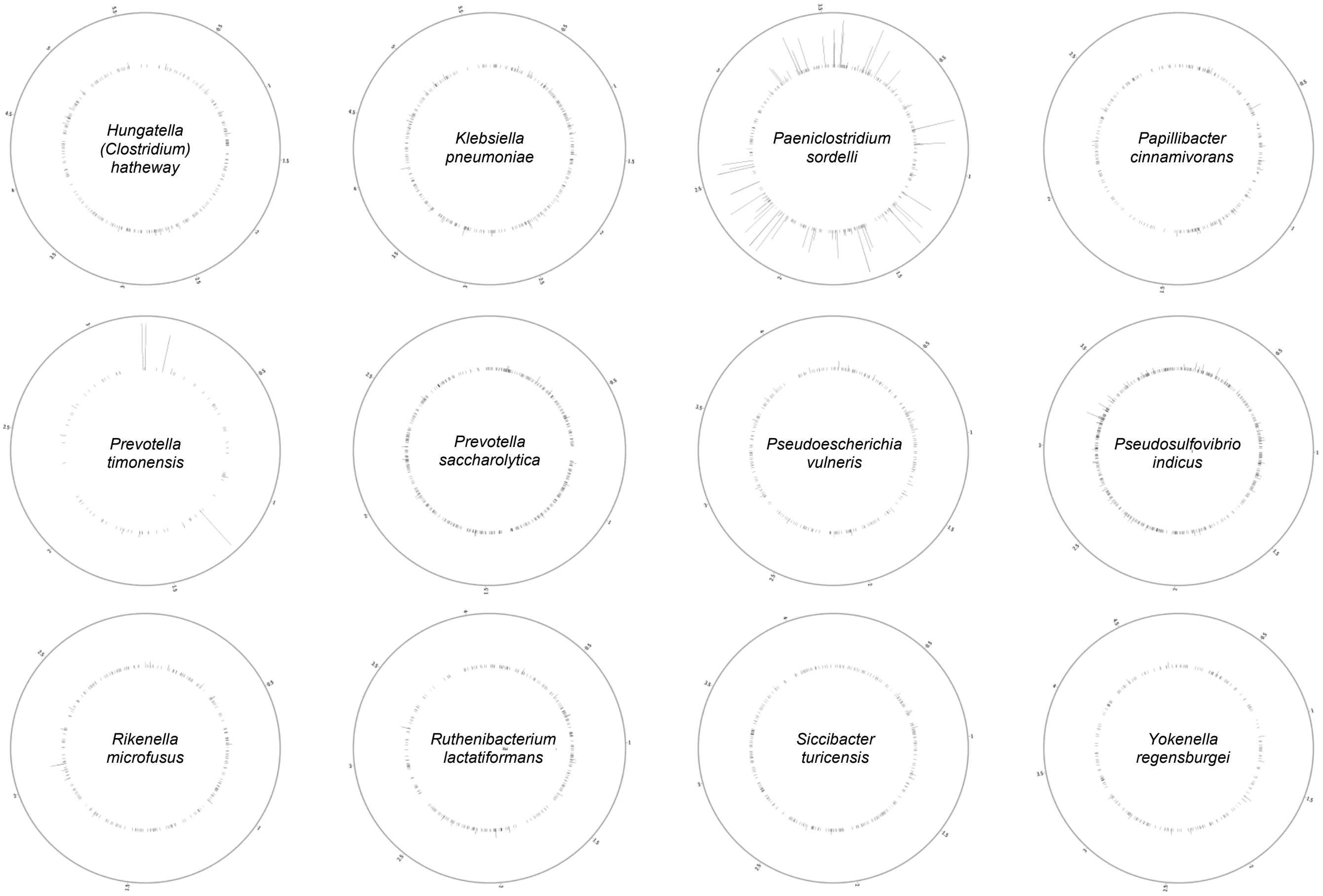

Supplementary Figure 7.
Coverage plots for bacterial taxa recovered from the El Salt sediments.

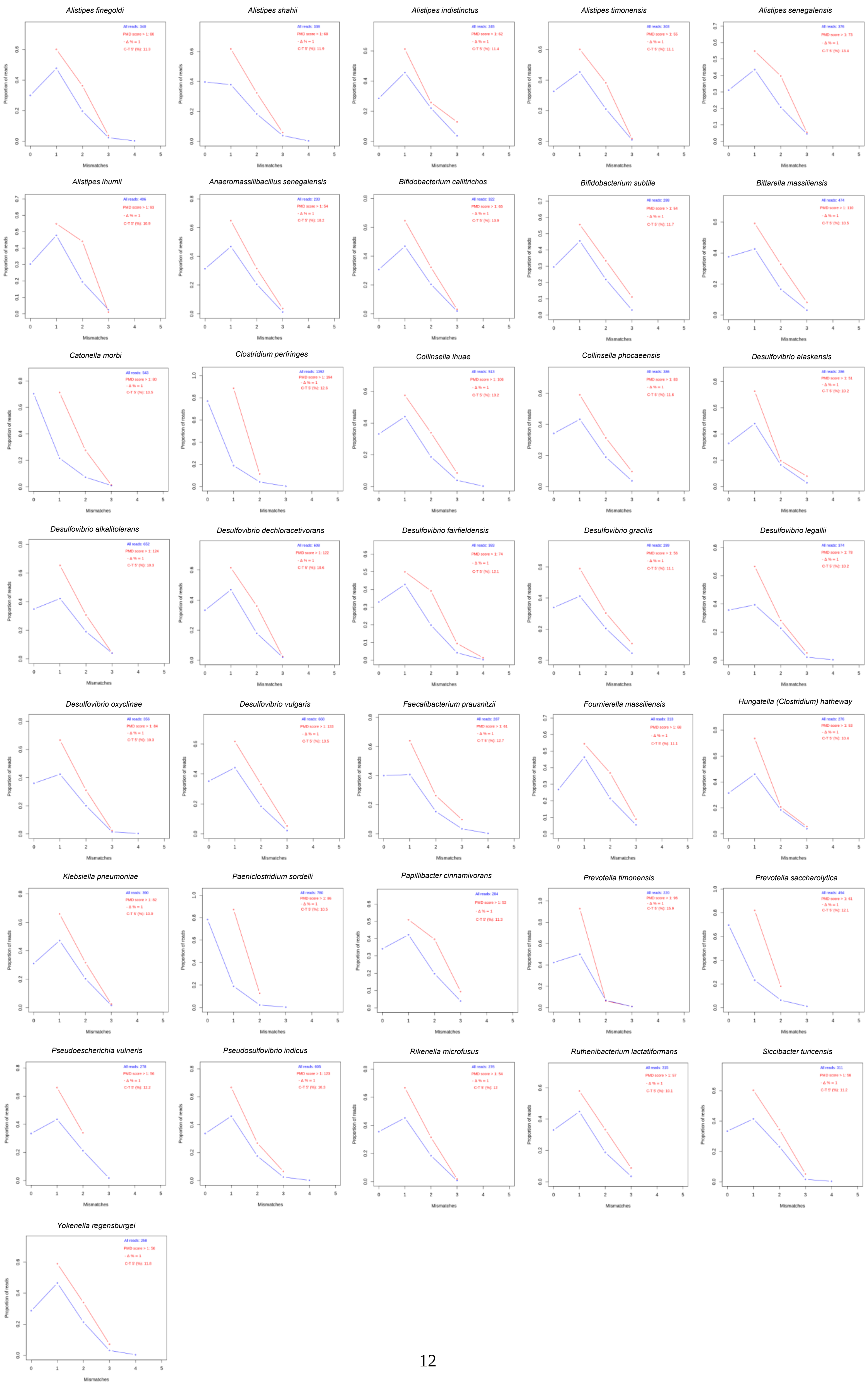
Alistipes finegoldi
Alistipes shahii
Alistipes indistinctus
Alistipes timonensis
Alistipes senegalensis
Alistipes ihumii
Anaeromassilibacillus senegalensis
Bifidobacterium callitrichos
Bifidobacterium subtile
Bittarella massiliensis
Catonella morbi
Clostridium perfringes
Collinsella ihuae
Collinsella phocaeensis
Desulfovibrio alaskensis
Desulfovibrio alkalitolerans
Desulfovibrio dechloracetivorans
Desulfovibrio fairfieldensis
Desulfovibrio gracilis
Desulfovibrio legallii
Desulfovibrio oxyclinae
Desulfovibrio vulgaris
Faecalibacterium prausnitzii
Fournierella massiliensis
Hungatella (Clostridium) hatheway
Klebsiella pneumoniae
Paeniclostridium sordelli
Papillibacter cinnamivorans
Prevotella timonensis
Prevotella saccharolytica
Pseudoescherichia vulneris
Pseudosulfovibrio indicus
Rikenella microfusus
Ruthenibacterium lactatiformans
Siccibacter turicensis
Yokenella regensburgei
Proportion of reads
Mismatches

Supplementary Figure 8.
Edit distance distributions of all reads (blue) and reads filtered for postmortem damage (PMDS>1) (red) for bacterial taxa with ≥200 assigned reads with >50 PMDS>1 reads recovered from the El Salt sediments.

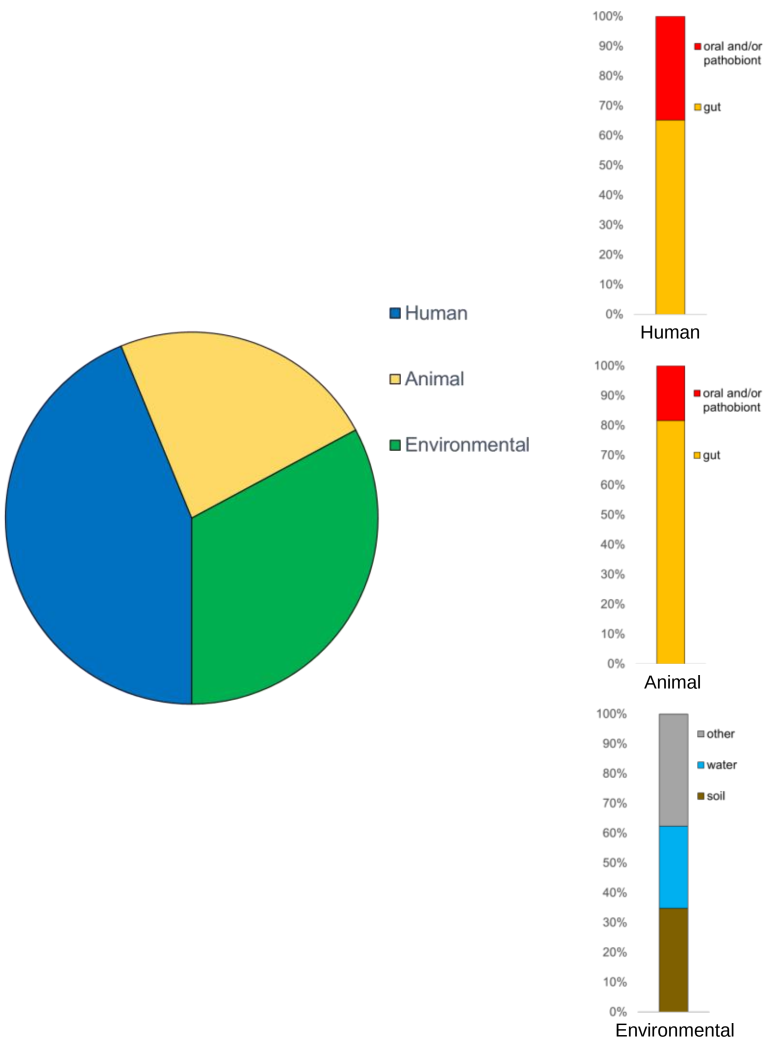

Supplementary Figure 9.
Major source categories for the bacterial species of hominid-associated gut microbiome families, as detected in El Salt sediment samples. Bacterial species were categorized according to a layered classification scheme as human (gut, oral and/or pathobiont), animal (gut, oral and/or pathobiont) and environmental (soil, water, other). Pie and bar charts show the proportion of species by source category.

Supplementary Table 1.
Number of ancient bacterial sequences identified in El Salt sediment samples. For each sample (V1 to XI) and negative control (ExtNeg, DNA extraction; LibNeg, library preparation; PCRNeg, PCR), the number of ancient bacterial sequences, as defined based on the approach by Skoglund *et al.* (2014), is shown, along with the number of paired-end sequences, pre and post-quality filtering.

Sample	Initial paired-end sequences	High-quality joined reads	Ancient bacterial reads
V1	7,451,006	3,666,150	1,422
V2	8,326,191	3,614,022	1,229
V3	5,215,587	2,369,988	1,725
IX	22,001,002	1,144,854	279
Xa	219,636,326	9,275,342	4,257
ES1	9,822,677	8,806,814	8,537
ES2	16,013,042	15,122,557	12,689
ES3	16,182,726	15,009,763	12,674
ES4	14,716,570	13,818,087	9,555
ES5	12,261,584	10,757,973	10,121
ES6	11,708,225	10,491,866	8,420
ES7	25,055,781	21,083,124	17,901
Xb	13,378,449	5,764,889	2,278
XI	7,590,144	3,667,077	4,615
ExtNeg	454,624	411,559	144
LibNeg	113,428	27,284	42
PCRNeg	2,597	2,263	1

Supplementary Table 2.
Ancient human mtDNA reads in the El Salt samples. All samples were subjected to target capture of mtDNA with a Neanderthal bait panel and subsequent NGS on Illumina NextSeq platform. Reads with a deamination profile resulting in PMDS >1 were used to calculate the breadth of coverage (>1x) and the negative difference proportion introduced by Hübler *et al.* (2019). Modern contamination refers to the results of the mtCont script of the Schmutzi pipeline (Renaud *et al.*, 2015). Such analysis was performed only for samples with breadth of coverage >10%. Samples with >1,000 PMDS>1 reads, breadth of coverage >10%, -Δ % ≥0.9 and mtCont contamination less than 2% were considered to contain ancient human mtDNA.

Sample	Reads	Reads PMDS >1	>1x (%)	-Δ %	Modern Contamination
V1	33,325	162	4.0	1	NA
V2	270,437	26,570	4.8	1	NA
V3	469,442	158	2.9	1	NA
IX	1,587	0	0.4	0	NA
Xa	1,669,396	962	2.0	1	NA
ES1	238,391	21,977	11.2	1	1%
ES2	1,143,745	24,041	27.6	1	1%
ES3	524,157	822	13.7	1	NA
ES4	239,272	244	6.7	1	NA
ES5	833,836	7,855	15.7	1	1%
ES6	38,176	1,153	6.9	1	NA
ES7	83,774	30	3.0	1	NA
Xb	112,244	1,830	19.1	1	2%
XI	16	0	0.0	0	NA

Supplementary Table 3.
Adapter and oligo sequences used in the present study.

**Name**	**Sequence 5'-3'**	**Use**	**Ref.**
IS1	A*C*A*C*TCTTTCCCTACACGACGCTCTTCCG*A*T*C*T	For all adapters	Meyer and Kircher, 2010
IS2	G*T*G*A*CTGGAGTTCAGACGTGTGCTCTTCCG*A*T*C*T	For all adapters	Meyer and Kircher, 2010
IS3_BEDC3	A*G*A*T*CGGAA*G*A*G*C[C3spacer]	For BEDC3 adapter	Carøe *et al.*, 2017
P7 index primer	CAAGCAGAAGACGGCATACGAGATNNNNNNNNGTGACTGGAGTTCAGACGTGTGCTCTTCCG	Index PCR	Present paper
P5 index primer	AATGATACGGCGACCACCGAGATCTACACNNNNNNNNACACTCTTTCCCTACACGACGCTCTTCCGATCT	Index PCR	Present paper

Supplementary References

1. Carøe, C. *et al.* Single-tube library preparation for degraded DNA. *Methods Ecol. Evol.* **9**, 410-419 (2018).
2. Hübler, R. *et al.* HOPS: automated detection and authentication of pathogen DNA in archaeological remains. *Genome Biol.* **20**, 280; 10.1186/s13059-019-1903-0 (2019).
3. Meyer, M. & Kircher, M. Illumina sequencing library preparation for highly multiplexed target capture and sequencing. *Cold Spring Harb. Protoc.* **2010**(6), pdb.prot5448 (2010).
4. Obregon-Tito, A. J. *et al.* Subsistence strategies in traditional societies distinguish gut microbiomes. *Nat. Commun.* **6**, 6505; 10.1038/ncomms7505 (2015).
5. Rampelli, S. *et al.* Metagenome sequencing of the Hadza hunter-gatherer gut microbiota. *Curr. Biol.* **25**, 1682-1693 (2015).
6. Renaud, G., Slon, V., Duggan, A. T. & Kelso, J. Schmutzi: estimation of contamination and endogenous mitochondrial consensus calling for ancient DNA. *Genome Biol.* **16**, 224; 10.1186/s13059-015-0776-0 (2015).
7. Skoglund, P. *et al.* Separating endogenous ancient DNA from modern day contamination in a Siberian Neandertal. *Proc. Natl. Acad. Sci. U.S.A.* **111**, 2229-2234 (2014).
