# Supplementary material for: Components of a Neanderthal gut microbiome recovered from fecal sediments from El Salt
Source: Commun Biol. 2021 Feb 5;4:169. doi: 10.1038/s42003-021-01689-y (PMC7864912; doi:10.1038/s42003-021-01689-y)
Supplement: Supplementary file 3 — Description of Additional Supplementary Files [file 42003_2021_1689_MOESM3_ESM.pdf]

Description of Additional Supplementary Files

File name: Supplementary Data 1
Description: List of bacterial species assigned to the ancient sequences retrieved in the negative controls. For each taxon, the read count in DNA extraction (ExtNeg), library preparation (LibNeg) and PCR (PCRNeg) negative controls is reported. No overlap was observed with the sample dataset.

File name: Supplementary Data 2
Description: Relative abundances of bacterial families detected across El Salt sediment samples. First row, number of reads with PMDS >5.

File name: Supplementary Data 3
Description: List of bacterial species identified in El Salt sediment samples and belonging to hominid-associated gut microbiome families. Only bacterial species from ancient sequences belonging to gut microbiome families shared among hominids (Bittar et al., 2014; Moeller et al., 2014; Zhang et al., 2015a; Lloyd-Price et al., 2016; Clayton et al., 2018; Hicks et al., 2018; Nishida et al., 2018, 2019), as in Fig. 4, are listed. For each species, the first documented source of isolation and the original reference are reported. When the bacterial species was not first isolated from feces or the intestinal tract, evidence of its presence in the human or animal gut was specifically sought.
