# Supplementary material for: Components of a Neanderthal gut microbiome recovered from fecal sediments from El Salt
Source: Commun Biol. 2021 Feb 5;4:169. doi: 10.1038/s42003-021-01689-y (PMC7864912; doi:10.1038/s42003-021-01689-y)
Supplement: Supplementary file 7 — Reporting Summary [file 42003_2021_1689_MOESM7_ESM.pdf]

nature research

Corresponding author(s): Marco Candela, Stephanie L. Schnorr

Last updated by author(s): Dec 27, 2020

Reporting Summary

Nature Research wishes to improve the reproducibility of the work that we publish. This form provides structure for consistency and transparency in reporting. For further information on Nature Research policies, see Authors & Referees and the Editorial Policy Checklist .

Statistics

For all statistical analyses, confirm that the following items are present in the figure legend, table legend, main text, or Methods section.

n/a	Confirmed	
☐	☒	The exact sample size (n) for each experimental group/condition, given as a discrete number and unit of measurement
☐	☒	A statement on whether measurements were taken from distinct samples or whether the same sample was measured repeatedly
☐	☒	The statistical test(s) used AND whether they are one- or two-sided<br>*Only common tests should be described solely by name; describe more complex techniques in the Methods section.*
☒	☐	A description of all covariates tested
☒	☐	A description of any assumptions or corrections, such as tests of normality and adjustment for multiple comparisons
☐	☒	A full description of the statistical parameters including central tendency (e.g. means) or other basic estimates (e.g. regression coefficient) AND variation (e.g. standard deviation) or associated estimates of uncertainty (e.g. confidence intervals)
☐	☒	For null hypothesis testing, the test statistic (e.g. F, t, r) with confidence intervals, effect sizes, degrees of freedom and P value noted<br>*Give P values as exact values whenever suitable.*
☒	☐	For Bayesian analysis, information on the choice of priors and Markov chain Monte Carlo settings
☒	☐	For hierarchical and complex designs, identification of the appropriate level for tests and full reporting of outcomes
☒	☐	Estimates of effect sizes (e.g. Cohen's d, Pearson's r), indicating how they were calculated

Our web collection on statistics for biologists contains articles on many of the points above.

Software and code

Policy information about availability of computer code

Data collection	No software was used
Data analysis	BWA aln algorithm; MEGAN utils package; samtools suite; QIIME; MUSCLE; FastTree; GraPhlAn; R software; MapDamage; bedtools; Circos; Schmutzi; PMDtools

For manuscripts utilizing custom algorithms or software that are central to the research but not yet described in published literature, software must be made available to editors/reviewers. We strongly encourage code deposition in a community repository (e.g. GitHub). See the Nature Research guidelines for submitting code & software for further information.

Data

Policy information about availability of data

All manuscripts must include a data availability statement. This statement should provide the following information, where applicable:

- Accession codes, unique identifiers, or web links for publicly available datasets
- A list of figures that have associated raw data
- A description of any restrictions on data availability

Sequences were deposited in the European Nucleotide Archive (ENA; project ID PRJEB41665).

Field-specific reporting

Please select the one below that is the best fit for your research. If you are not sure, read the appropriate sections before making your selection.

☐ Life sciences ☐ Behavioural & social sciences ☒ Ecological, evolutionary & environmental sciences

Ecological, evolutionary & environmental sciences study design

All studies must disclose on these points even when the disclosure is negative.

Study description	Ancient human gut microbiota components were identified by shotgun metagenomics analysis of ancient DNA extracted from archeological sedimentary samples from different stratigraphic units of the Middle Paleolithic open-air site, El Salt (Alicante, Spain).
Research sample	14 archeological sediments spanning four stratigraphic units of El Salt Middle Paleolithic site (Spain), including layers of unit X, which has yielded well-preserved Neanderthal occupation deposits dating around 50 kya.
Sampling strategy	The samples were collected from different zones of the current El Salt excavation area, poor or rich in archaeological remains.
Data collection	C.M., C.H., B.G., and A.S. performed the field work, excavation, and sampling. Loose sediment samples (5-10 g) were collected in plastic vials using sterilized spoons (one per sample) after thoroughly cleaning the excavation surface with a vacuum cleaner in order to guarantee removal of any recent dust or sediment blown in from a different location. Lab safety masks and nitrile gloves were used at all times.
Timing and spatial scale	ES1 to ES7 samples were collected from the stratigraphic unit (SU) X (subunit Xb-H44) before 2014 (please, see Sistiaga et al., 2014). The present work also includes an additional seven new archeological sediments collected in 2018 as a control, from SU X (two samples from subunit Xa and Xb, respectively) and surrounding SUs, i.e. upper V (three samples), IX and XI (one sample each).
Data exclusions	No data were excluded from the analyses.
Reproducibility	Multiple samples were taken per stratigraphic unit.
Randomization	Samples were stratified based on the archaeologicla record.
Blinding	The objective was to identify ancient human gut microbiome components in samples rich in archaeological remains as compared to those archaeologically poor so blinding was not possible.

Did the study involve field work? ☒ Yes ☐ No

Field work, collection and transport

Field conditions	The samples were collected from different zones of the current El Salt excavation area, during summer 2018.
Location	The samples were collected from:<br>1) Zone 1. This is the upper excavation zone. Samples were collected from stratigraphic unit (SU) V, Facies 23 (one sample, V1) and Facies 24 (two samples, V2 and V3). This unit has been dated by OSL to 44.7 ± 3.2 ky BP.<br>2) Zone 2. This is the lower excavation zone. Samples were collected from SUs IX, Xa, Xb and XI, which are a stratified succession of sedimentary layers rich in Middle Paleolithic archaeological remains. From top to base:<br>- Unit IX (one sample): is the uppermost layer in this succession.<br>- Unit Xa (one sample): dated by TL to 52.3 ± 4.6 ky BP.<br>- Unit Xb (eight samples): similar to Xa. Seven samples from this unit (ES1-7) were collected from a microstratified combustion structure (H44) at the top of this layer that yielded human fecal biomarkers. The other sample was collected from underlying sediment.<br>- Unit XI (one sample).
Access and import/export	Samples were collected from the current El Salt excavation area and exported in a respnsible manner and in compliance with local, national and international laws. Archaeological research at El Salt is funded by Spanish I+D Project HAR2008-06117/HIST (C.M., C.H., B.G.).
Disturbance	The study did not cause any disturbance.

Reporting for specific materials, systems and methods

We require information from authors about some types of materials, experimental systems and methods used in many studies. Here, indicate whether each material, system or method listed is relevant to your study. If you are not sure if a list item applies to your research, read the appropriate section before selecting a response.

Materials & experimental systems

n/a	Involved in the study
☒	☐ Antibodies
☒	☐ Eukaryotic cell lines
☒	☐ Palaeontology
☒	☐ Animals and other organisms
☒	☐ Human research participants
☒	☐ Clinical data

Methods

n/a	Involved in the study
☒	☐ ChIP-seq
☒	☐ Flow cytometry
☒	☐ MRI-based neuroimaging
